# A Functional Assessment Tool to Distinguish Controls From Alzheimer’s Disease in Lima, Peru

**DOI:** 10.1177/15333175221104354

**Published:** 2022-06-03

**Authors:** Nilton Custodio, Rosa Montesinos, Diego Chambergo-Michilot, Eder Herrera-Perez, Maritza Pintado-Caipa, Wendy Seminario G, José Cuenca, Laura Mesía, Virgilio E Failoc-Rojas, Monica M Diaz

**Affiliations:** 1 Servicio de Neurología, Instituto Peruano de Neurociencias, Lima, Perú; 2 Unidad de Diagnóstico de Deterioro Cognitivo y Prevención de Demencia, Instituto Peruano de Neurociencias, Lima, Perú; 3 Unidad de Investigación, Instituto Peruano de Neurociencias, Lima, Perú; 4 Escuela Profesional de Medicina Humana, Universidad Privada San Juan Bautista, Lima, Perú; 5 Servicio de Rehabilitación, Instituto Peruano de Neurociencias, Lima, Perú; 6 Universidad Científica Del Sur, Lima, Perú; 7 Grupo de Investigación Molident, 33225 Universidad San Ignacio de Loyola, Lima, Perú; 8 Atlantic Fellow for Equity in Brain Health, Global Brain Health Institute, University of California, San Francisco, San Francisco, CA, USA; 9 Servicio de Neuropsicología, Instituto Peruano de Neurociencias, Lima, Peru; 10 Carrera de Psicología, Facultad de Ciencias de La Salud, Universidad Privada Del Norte, Lima, Perú; 11 Salud Mental, Universidad Privada Norbert Wiener, Lima, Perú; 12 Department of Neurology, University of North Carolina at Chapel Hill, Chapel Hill, NC, USA; 13 Facultad de Salud Pública y Administración, Universidad Peruana Cayetano Heredia, Lima, Perú

**Keywords:** alzheimer disease, dementia, neurocognitive disorders, peru, validation study

## Abstract

**Background:**

The Alzheimer’s Disease Cooperative Study-Activities of Daily Living (ADCS-ADL) scale is a versatile functional assessment tool for patients with Alzheimer’s disease (ad). We evaluated its performance in controls, Peruvians with MCI or AD.

**Methods:**

A cross-sectional study of older adults attending a neurology institute in Lima (Peru) with mild cognitive impairment (MCI), ad or cognitively healthy. Test-retest reliability (intraclass correlation coefficient, ICC; internal consistency, Cronbach’s alpha) and validity were assessed.

**Results:**

We enrolled 276 individuals (ad: 113, MCI: 68, controls: 95) with no age, sex, educational level, and depressive symptom differences. Reliability was ideal (ICC: .996), and Cronbach’s alpha was adequate (.937). The ADCS-ADL could not differentiate MCI from controls but did differentiate ad severity. The ADCS-ADL correlated highly with nearly all tools.

**Conclusions:**

The ADCS-ADL scale is reliable in a population with ad in Lima, Peru. Future work may validate a tool for Peruvians with lower educational levels.

## Introduction

Alzheimer’s disease (ad) is a progressive cognitive impairment with predominant memory impairment (although non-amnestic presentations exist) that may or may not be accompanied by psychological or behavioral symptoms. One of the most debilitating features of ad is the functional impairment that occurs as the disease progresses, impeding ability to maintain work, social or family obligations reflected in a decline of activities of daily living (ADLs) and functional performance.^1^ Mild cognitive impairment (MCI), a pre-cursor to ad, is defined as having impairment in 1 or more cognitive domains while maintaining functional (instrumental) independence.^2^ The feature distinguishing these 2 entities is the preservation of function (ie the ability to perform ADLs).^1,2^ ADLs include Cognitive ADLs (C-ADLs),^3^ defined as functions that rely on the executive function domain, such as one’s ability to manage finances, perform social planning or complex verbal activities (ie reading and writing). Instrumental ADLs (I-ADLs) are ADLs that require managing complex activities, such as proper use of household appliances (ie using the telephone, operating a television or household appliances), completing household chores (ie cooking or meal preparation, taking out the trash).^1,4^ Basic ADLs (B-ADLs) are a set of primary activities that a person must complete to maintain basic autonomy and independence without relying on others, such as feeding, toileting and dressing (4-6). Despite this, all types of ADLs require cognition, and can be classified as cognitive, instrumental or basic ADLs. This classification is used to differentiate between types of activities the person is able to complete. For example, patients with MCI may notice difficulties performing C-ADLs,^4^ while patients with early-stage ad may have difficulties with I-ADLs. In more advanced stages of ad, B-ADLs are compromised as the disease progresses leading to total dependence on another person to complete these B-ADLs, usually a family member or caretaker.^5-8^

Because functional assessment is required to distinguish between MCI and stages of ad and to make an appropriate diagnosis, development of brief functional assessment screenings are needed that are validated for different cultural contexts. Several functional assessment scales have been developed to measure ADL capacities of patients with dementia, and some have been validated for use in Latin American populations. For example, the Pfeffer Functional Activities Questionnaire (PFAQ), validated to detect dementia by Quiroga et al in Chile^9^ is a simple questionnaire designed for community studies in normal individuals or with atypical cognitive alterations. The questionnaire is administered to an informant familiar with the patients’ day-to-day activities, measures the patient’s functional ability to perform I-ADLs and correlates highly with cognitive impairment severity. The PFAQ correlates highly (r = .76) with cognitive function and is able to distinguish between cognitively healthy individuals and subjects with depression (pseudo-dementia) or dementia.^10^ The PFAQ also correlates highly (r = .72) with another widely used scale, the Lawton and Brody^11^’s Instrumental Activities of Daily Living Scale, for patients with dementia. In Peru, the PFAQ is the only tool that has been utilized to assess functionality, and has also been used in Chile^9,12^ and Brazil.^13^

The Alzheimer’s Disease Cooperative Study-Activities of Daily Living (ADCS-ADL) scale was designed for ADL assessment in clinical trials of patients with AD.^14^ The ADCS-ADL is a scale that assesses 23 B-ADLs and I-ADLs. Each activity is scored ranging from 0 (patient does not perform the activity) to the highest score (patient is independent in the activity). The ADCS-ADL has several advantages, including ease of administration by primary health care providers and that it is able to monitor functional decline using longitudinal assessments of patients with AD.^15^ Moreover, it is important to culturally and linguistically validate a tool for a particular country, given cross-cultural differences that may bias the validity of the original tool. In Peru, the prevalence of AD and other related dementias is as high 520 people per 100,000.^16^ This number may increase further in high-altitude areas compared with lower altitude areas, many of which exist in Peru.^17^ Despite the need to reduce the burden of AD, few validated scales, such as the RUDAS tool, exist for dementia screening in Peru.^18^ Given the large burden of AD patients,^19^ validation of shorter screening tools for quicker identification of these patients in the primary care setting, are needed, such as the ADCS-ADL.

Our study aimed to evaluate the performance of the ADCS-ADL in cognitively healthy controls and a population of Peruvian patients with a diagnosis of MCI or AD and to explore its validity and reliability in measuring ADLs in this population. We hypothesized that ADCS-ADL has acceptable performance to assess functional status in controls, patients with MCI and AD in a Peruvian population. Therefore, we sought to test the psychometric properties of the ADCS-ADL to distinguish between cognitively healthy controls, MCI and AD patients and to determine the test’s sensitivity in this population.

## Methods

### Study Participants

This was a cross-sectional study that included randomly-selected individuals who were classified into 3 groups: ad, MCI and cognitively healthy participants. All MCI and AD patients attended the Cognitive Impairment and Dementia Diagnosis and Prevention Unit of the *Instituto Peruano de Neurociencias* (IPN) between January 2015 and December 2020 located in Lima, the capital city of Peru. Inclusion criteria were: male and female individuals over 60 years of age who met the diagnostic criteria for AD, MCI, or cognitively healthy individuals as defined below. The diagnosis of probable AD was made according to the National Institute of Aging (NIA) and the Alzheimer’s Association (AA) criteria,^20^ and a diagnosis of MCI was made according to criteria established by the NIA-AA.^21^ The Clinical Dementia Rating (CDR) scale was applied to determine severity or stage of AD (mild, moderate, or severe).^22^ The cognitively healthy control group consisted of patients’ relatives or healthy volunteers without cognitive complaints. They had normal results in the following screening tools: the Mini-Mental State Examination (MMSE), the Clock Drawing Test (CDT) and the PFAQ. Exclusion criteria included individuals: (1) with difficulty performing cognitive tests due to hearing, visual or other physical problems that would interfere with their performance on the tests; (2) with a primary language other than Spanish; (3) with low educational level, defined as an individual with less than 4 years of education; individuals with a current diagnosis of depression (including those on antidepressants); (4) with a history of addiction or substance abuse; (5) with vascular dementia, frontotemporal dementia, Parkinson’s disease with dementia or Dementia with Lewy bodies; (6) with cognitive impairment explained by another cause, such as hypothyroidism, vitamin B12 deficiency, liver disease, chronic kidney disease, neurological infections (ie HIV-associated opportunistic infections, syphilis), severe head trauma, subdural hematoma, among others.

### Ethical Considerations

All participants and their caregivers signed an informed consent form in accordance with the ethical guidelines for research on human subjects. The study protocol was approved by the ethics committee of the Universidad de San Martin de Porres (approval number 63-2014).

### Functional Assessment Tools

#### Alzheimer´s Disease Cooperative Study-Activities of daily Living (ADCS-ADL)

The ADCS-ADL is a functional assessment scale that evaluates various ADLs specifically for patients with AD. The original version includes 23 questions, 7 of which ask about B-ADLs and 17 questions assess I-ADLs. The questionnaire is provided to caregivers (ie spouse, children, hired caregivers or informal caregivers who report caring for the patient for more than 6 hours per day, during the 3 consecutive months prior to the assessment). The informant is asked about activities performed by the patient in the 4 weeks prior and the extent to which the patient could perform those activities by themselves without help. Each of the items is scored ranging from complete independence to total dependence. The total score is obtained by the sum of the 23 individual items and scores range between 0 to 78 points, where the lowest value indicates total dependence.^11^

#### Clinical Dementia Rating (CDR)

The CDR is a widely-used 5-point scale that evaluate 6 domains (memory, orientation, judgment and problem solving, ability to work in the community, ability to complete daily tasks at home, hobbies and self-care). The CDR was used to grade the severity of dementia among participants. Those in the cognitive healthy control group had a score of “0" (no dementia). Those with in the MCI group had a CDR score of .5 (“suspected dementia” or “questionable dementia”). Those in the ad group had CDR scores of 1, 2 or 3 representing mild, moderate or severe stages of ad, respectively.^23^

#### Clinical and Neuropsychological Evaluations

Individuals attending the Cognitive Impairment and Dementia Diagnosis and Prevention Unit of the IPN underwent the following sequential assessments in each phase: (a) screening, (b) dementia diagnosis and (c) determination of dementia type. The screening phase lasted 20 minutes, and the diagnostic phase 40 minutes. Both of phases were performed during an outpatient visit by neurologists and a specialist in neurorehabilitation. Next, in the determination of dementia type phase, neuropsychologists tested patients for 2 hours as described below.

### Screening Phase

During the screening phase, individuals underwent a comprehensive clinical assessment and brief cognitive tests, which included the MMSE,^24^ CDT-Manos version^25^ and Pfeffer Functional Activities Questionnaire (PFAQ).^9^ Those individuals obtaining scores below the cut-off for cognitive impairment for this study protocol underwent a second evaluation, in which a second MMSE and CDT-Manos version were administered by a different evaluator than the 1 who performed the first administration during the screening phase.

The cut-off score for suspected dementia on the MMSE was adjusted according to years of education: 27 for individuals with more than 7 years of education and 23 for those with 4 to 7 years of education.^24^ The CDT-Manos version assesses an individual’s ability to draw a clock accurately, and then assesses the direction and proportionality of the clock’s hands as the patient attempts to indicate the time at 11:10 o’clock. The maximum score is 10, and in Peruvian individuals a score below 7 indicates cognitive impairment.^25^ The PFAQ includes 11 questions about activities of daily living, with score ranging from 0 to 3 according to the functional disability severity for each activity assessed. The maximum score is 33, and a score greater than 6 indicates functional impairment.^9^

### Dementia Diagnosis Phase

Individuals who in the screening phase were confirmed to have “cognitive impairment” underwent a blood draw (hemoglobin levels, glucose, urea, creatinine, liver function tests, vitamin B12 and folic acid levels, thyroid profile (T3, T4 and thyroid stimulating hormone), serum electrolyte levels (sodium, potassium, chloride); VDRL to rule out syphilis and HIV ELISA to rule out HIV. Neuroimaging was performed at this phase with brain CT scan and/or brain MRI. A depression screening scale was administered using the Beck Depression Inventory-II (BDI-II) to rule out pseudo-dementia^26^ CDR, and the Global Deterioration Scale (GDS),^27^ Addenbrooke’s Cognitive Examination (ACE)^28^ and Alzheimer’s Disease Assessment Scale-Cognitive subscale (ADAScog)^29^ scales were applied.

The ACE is a 15 to 20 minute cognitive test that has been validated for use in Peru that evaluates 6 cognitive domains. The maximum score is 100: orientation [10 points], attention [8 points], memory [35 points], verbal fluency [14 points], language [28 points] and visuospatial skills [5 points](28). The Alzheimer’s disease Assessment Scale (ADAS) is an instrument designed to assess cognitive impairment severity (ADAScog) and non-cognitive (ADASnocog) impairments in patients with AD. The ADAScog consists of 11 items, with scores ranging from 0 (no impairment) to 70 (severe dementia). The cognitive function sections evaluates memory (27 points), orientation (8 points), language (25 points) and praxia (10 points), and has been validated in Spanish.^30^

### Determination of Dementia Type Phase

In the last phase, a study neuropsychologist performed a neuropsychological evaluation on each patient including the following batteries: Rey Auditory Verbal Learning Test,^31^ Logical Memory Subtest of the revised Weschler Memory Scale,^32^ Trail Making Test A and B,^33^ Rey-Osterrieth complex figure,^31^ Boston naming test,^34^ Wisconsin Card Sorting Test,^35^ Letter-Number (subtest of the Weschler Adult Intelligent Scale III), Digit Span, Strub-Black Mental Status drawing test, WAIS-III cubes test.^32^ Finally, another neuropsychologist applied the neuropsychiatric battery of the Neuropsychiatric Inventory^36^ and a functional evaluation using the ADCS-ADL,^14^ and the Instrumental Activities of Daily Living (IADL) and the Basic Activities of Daily Living (BADL) Questionnaire.^37^ The IADL consists of 7 items: telephone use, travel, shopping, meal preparation, housework, taking own medicine, and handling personal finances. The BADL includes self-maintenance skills such as dressing, bathing, and grooming.

Using a composite report based on the neuropsychological, neuropsychiatric and functional evaluations and the blood test and neuroimaging results, diagnosis of the type of dementia was made by consensus between neurologists and neuropsychologists of the study team. The neuropsychologists who applied the neuropsychological, neuropsychiatric and functional batteries were unaware of the results of the brief cognitive tests and dementia severity scales performed in the screening and dementia diagnosis phase.

### Statistical Analyses

Demographic variables were compared between groups (ad vs controls; ad vs MCI; MCI vs controls) using One-Way Analysis of Variance (ANOVA) with Bonferroni post hoc corrections when necessary. For categorical variables, proportions were compared using Chi-square tests. The psychometric properties of the ADCS-ADL that were analyzed included:

(1) Test-retest reliability of the ADCS-ADL was determined using the intra-class correlation coefficient (ICC) and internal consistency using Cronbach’s alpha coefficient. The Cronbach’s alpha coefficient indicates degree of consistency (variance) between the ADCS-ADL items ranging from .0 to 1.0. A good internal consistency is considered a Cronbach’s alpha value closer to 1.0. Cronbach’s alpha was also used to evaluate item removal from the ADCS-ADL, such that the alpha coefficient was re-calculated each time an item was removed from the scale. If the alpha value obtained after removing an item from the scale was high, this indicated that the individual item provided a negative contribution to internal consistency indicating it was not adequate for functional assessment in our population. If both ICC and Cronbach’s alpha were closer to 1.0, the tool was considered reliable.
(2) Validity of the ADCS-ADL was assessed by measuring external validity (sensitivity) and convergent validity. The external validity of the ADCS-ADL was assessed using the CDR, the dementia staging scale. The CDR levels (.5, 1, 1, 2 and 3) were used as criteria for dementia severity. The ADCS-ADL scores of each group (ad, MCI and controls) were compared with the CDR scores to determine the sensitivity of the ADCS-ADL scores to accurately determine dementia severity. Convergent validity was determined by comparing ADCS-ADL total scores using the Fillenbaum scale (IADL and BADL),^37^ MMSE, ACE, CDR, GDS and ADAScog total score by applying Spearman correlation coefficients. Logistic regression (logit) was performed for each pair of study groups (ad/MCI, MCI/control and ad/control) using a two-variable model: final diagnosis (control, MCI or ad) as dependent variables and each cognitive test (ADCS-ADL, Fillenbaum scale (BADL, IADL), MMSE, ACE, ADAScog, CDR and GDS) as independent variables.

## Results

There were 276 individuals included in the analysis categorized into 3 study groups: 95 cognitively healthy controls, 68 with a diagnosis of MCI and 113 with a diagnosis of AD. There were no significant difference between the 3 groups (AD, MCI or cognitively healthy) in age, sex, educational level and BDI-II score. The MMSE was able to discriminate controls from patients with AD, but not cognitively healthy controls from patients with MCI. We found that the MMSE was able to discriminate between mild, moderate and severe stages of AD (Table 1).

**Table 1.** Demographic characteristics of controls, mild cognitive impairment and Alzheimer’s disease groups by Clinical Dementia Rating (CDR) scale scores.

	Cognitively Healthy Controls	MCI	Alzheimer’s Disease			
—	CDR 0 (n = 95)	CDR .5 (n = 68)	CDR 1 (n = 39)	CDR 2 (n = 38)	CDR 3 (n = 36)	*P*-value
Age	69.6 (7.9)	72.2 (7.3)	69.8 (8.9)	71.2 (9.3)	74.5 (8.1)	.09
Sex (F:M)	28:22	11:7	12:9	12:8	10:7	.36
Educational level (years)	11.7 (2.8)	11.4 (2.9)	12.1 (2.5)	12.3 (2.4)	11.8 (3.2)	.39
BDI-II	4.66 (2.6)	4.42 (2.3)	3.15 (2.2)	4.53 (2.1)	4.37 (2.5)	.41
MMSE	28.7 (.7)	27.6 (1.2)	22.1 (2.3)	16.8 (2.1)	8.6 (2.1)	< .001^a^
ACE	93.3 (2.6)	89.6 (4.6)	80.2 (2.3)	69.8 (3.7)	49.4 (3.2)	< .001^a^

CDR: Clinical Dementia Rating; BDI-II: Beck Depression Inventory-II; MMSE: Mini Mental State Examination; ACE: Addenbrooke´s Cognitive Examination.

^a^Control vs AD *P* < .001.

The test-retest reliability of the ADCS-ADL assessed by the ICC was ideal (.996, confidence interval [CI] .995-.998) for the study population. The internal consistency of the ADCS-ADL was satisfactory by comparing the Cronbach’s alpha coefficient of the ADCS-ADL of the AD group (CDR1 + CDR2 + CDR3 = 113) with the Cronbach’s alpha coefficient obtained for the ADCS-ADL scores of all groups studied (controls + MCI + AD [CDR1 + CDR2 + CDR3] = 276) (Table 2). The alpha coefficients obtained each time an item was removed indicated that item 2 of the ADCS-ADL (“walking”) was not adequate for the assessment, given an increase, or improvement, in the alpha coefficient when this item was removed during the analysis. In addition, the total correlation coefficients showed that items 2 (“walking”), 20 (“reading”) and 22 (“entertainment” or “hobbies”) correlated poorly with the total scale score.

**Table 2.** Internal consistency and correlations of the ADCS-ADL item-to-total scores for patients with ad

	AD (n = 113)	All Groups (n = 276)	Item-to-total Score Correlations in Patients with AD (n = 113)
Cronbach’s alpha coefficient	.937	.962	—
Chronbach’s alpha when each item is eliminated			—
Item 1: Eating	.936	.961	.68
Item 2: Walking	.938	.964	.38
Item 3: Toileting	.935	.961	.71
Item 4: Bathing	.932	.957	.88
Item 5: Grooming	.934	.961	.76
Item 6: Dressing self	.935	.960	.88
Item 7: Telephone	.935	.959	.67
Item 8: Television	.935	.960	.66
Item 9: Conversation	.933	.960	.79
Item 10: Dishes	.935	.960	.68
Item 11: Personal belongings	.935	.960	.66
Item 12: Prepare drinks	.933	.959	.73
Item 13: Prepare food or cooking	.935	.960	.67
Item 14: Throw out trash	.933	.959	.77
Item 15: Travel outside the home	.934	.958	.73
Item 16: Shopping	.935	.960	.64
Item 17: Keeping appointments	.934	.959	.68
Item 18: Stay alone at home safely	.935	.960	.65
Item 19: Familiarity with current events	.933	.959	.77
Item 20: Reading	.937	.962	.39
Item 21: Writing	.935	.958	.67
Item 22: Hobbies	.937	.962	.32
Item 23: Using home appliances	.936	.960	.71

AD: Alzheimer’s disease.

The validity or sensitivity of the ADCS-ADL was assessed using CDR stages. There were no significant differences between patients with MCI (CDR .5) and healthy controls. As CDR stages worsen, ADCS-ADL scores decreased significantly. Similar trends were observed with IADL and BADL scores (Table 3). However, the ADCS-ADL could not differentiate patients with MCI (CDR .5) from cognitively healthy controls (CDR 0), but it could accurately differentiate patients with MCI from those mild AD (CDR 1), moderate AD (CDR 2), or severe AD (CDR 3). The BADL could differentiate between mild, moderate and severe forms of AD, but it cannot differentiate controls from MCI and mild AD. The IADL scale could distinguish between cognitively healthy controls and patients with MCI, mild AD or moderate AD, but was unable to differentiate moderate AD from severe AD.

**Table 3.** Sensitivity of the ADCS-ADL, IADL and BADL scores by CDR stage in cognitively healthy controls, Mild Cognitive Impairment and Alzheimer’s Disease.

	Control	MCI	Alzheimer’s Disease			—
—	CDR 0	CDR 0.5	CDR 1	CDR 2	CDR 3	*P*-value^a^ (post hoc)^b^
ADCS-ADL	73.4 (4.6)	69.6 (6.8)	52.1 (9.6)	34.3 (10.6)	17.8 (10.2)	(CDR 0=CDR .5)>1>2>3
BADL	.03 (.2)	.2 (.6)	1.7 (.87)	3.7 (2.6)	7.5 (4.3)	(CDR 0=CDR .5=1)<2<3
IADL	.28 (.90)	1.8 (1.7)	10.2 (1.9)	12.7 (1.2)	14.2 (.01)	CDR 0<CDR .5<1<(2=3)

CDR: Clinical Dementia Rating; ADCS-ADL: Alzheimer’s Disease Cooperative Study – Activities of Daily Living; BADL: Basic Activities of Daily Living; IADL: Instrumental Activities of Daily Living.

^a^Kruskal Wallis ANOVA (*P* < .0001).

^b^Bonferoni (*P* = .015).

The convergent validity of the ADCS-ADL compared with the functional scales of activities of daily living (BADL, IADL) scale, cognitive scales (MMSE, ACE and ADAScog), and dementia severity scales (CDR and GDS) were performed (Table 4). The ADCS-ADL correlated highly with all scales except the ADAScog.

**Table 4.** Convergent validity^a^ of the ADCS-ADL compared with the BADL, IADL, MMSE, ACE, ADAScog, CDR and GDS scales for all study groups.

	BADL	IADL	MMSE	ACE	ADAScog	CDR	GDS
ADCS-ADL	.825	.826	.726	.839	.191	.828	.754

ADCS-ADL: Alzheimer’s Disease Cooperative Study – Activities of Daily Living; BADL: Basic Activities of Daily Living; IADL: Instrumental Activities of Daily Living; MMSE: Mini Mental State Examination; ACE: Addenbrooke´s Cognitive Examination; ADAScog: Alzheimer´s Disease Assessment Scale-cognitive subscale; CDR: Clinical Dementia Rating; GDS: Global Deterioration Scale.

^a^Spearman’s Rho.

## Discussion

Our study found an AD prevalence of 40.9% in this private multidisciplinary neurlogy institute in Lima, Peru. Importantly, this number does not represent the overall prevalence of the general Peruvian population since our study was carried out in a healthcare center for patients with neurological disorders. This prevalence is comparable to a prior study also completed in a private neurology institute in Brazil.^38^ Moreover, our study found that the MMSE is able to discriminate AD severity classifications, which has previously been reported in literature.^39^

Our study has shown adequate reliability of the ADCS-ADL scale (ICC: .996) in people with AD in an older adult population who receive medical care at a specialized neurology institute in Lima, Peru. Furthermore, this scale correlates highly with results of the most common functional assessment and cognitive screening scales applied to patients with AD. The high ICC and Cronbach’s alpha coefficient demonstrates that the ADCS-ADL is a reliable scale in our population. Test-retest consistency of the ADCS-ADL was found to be high (ICC: .996 for total score). In comparison, κ values were reported between .40-.75 for subscale scores in the original study.^14^

Similar to our study, 1 cultural adaptation study of the ADCS-ADL scale also demonstrated a high Cronbach’s alpha coefficient,^13^ indicating that the scale is accurate likely because the items correlate to a high degree. However, the items that assessed the ADLs of walking, reading and entertainment (hobbies) had a negative contribution to the internal consistency of the ADCS-ADL in our population. Since these specific ADLs become impaired in more advanced stages of AD, it is possible that a larger sample of patients with severe AD may be needed to determine if there may be greater consistency for these scale items. Galasko et al demonstrated that the ADCS-ADL items have adequate individual sensitivity for functional impairment in AD, however, its sensitivity may vary with severity of dementia.^14,40^ Another potential reason for the lack of internal consistency of these items may be that the question directionality may not be appropriate for severe AD. Therefore, it is necessary to assess not only the clinical impact of the individual questions and answers in patients with ad, but also the patient’s understanding of his or her disease.

The ADCS-ADL has been frequently used as an outcome measure in clinical trials of patients with AD,^40^ and has even been chosen as the ‘gold standard’ in other scale validation studies given its adequate psychometric properties in AD populations.^41^ Although the ADCS-ADL was found to be useful in our population of patients with AD, we found that it could not differentiate between MCI and controls, but it was able to differentiate between degrees of AD severity. The clinical implications of our findings suggest that the ADCS-ADL scale should not be used to screen for AD, but rather for follow-up assessments of patients with an established diagnosis of AD in clinical practice.

I-ADLs are the most sensitive of the ADLs to identify cognitive impairment and dementia given the first symptoms of MCI or early are usually memory deficits.^42^ As the disease progresses, B-ADLs become involved and assessment of B-ADLs are more specific to AD.^43^ Decline in I-ADLs is associated with hypometabolism in the inferior parietal, inferior temporal and superior occipital lobes.^8^ In patients with AD, total and individual neuronal synaptic density of the frontotemporal cortex decreases within 2 to 4 years after symptom onset.^42^ This demonstrates that functional assessment tools may also be useful for prognostication of the disease for patients and family members and caretakers.

In Peru, a low-to-middle income country in South America, the incidence of AD is high, exceeding 75 cases per 100,000 people according to the Global Burden of Disease metrics.^16^ Despite these numbers, there are insufficient functional assessment tools in AD validated for use in Peruvian populations. Therefore, it is necessary to validate a detailed functional battery, such as the ADCS-ADL, specific to the Peruvian context. The ADCS-ADL is useful as it covers several I-ADLs and B-ADLs, but it does have some limitations with longer administration times compared with other validated brief functional assessment tools, such as the PFAQ (15 min ADCS-ADL vs 3 min PFAQ).^13^ The length of time required to administer the ADCS-ADL may pose some limitations for its application in a primary health care center where a quick screening tool is needed to help guide appropriate patient referrals to a specialized center for patients with dementia. In addition, the IADL scale distinguished between controls, MCI, mild and moderate AD patients, highlighting it may be a better tool compared with the ADCS-ADL. However, given the ADCS-ADL covers more IADLs and BADLs compared with the PFAQ, it may serve as a more detailed longitudinal assessment of functional decline in those with an existing diagnosis of AD in Peruvian populations.

### Limitations

Our study has some limitations. First, the sampling was not random, which decreases the reliability of results. Next, patients were recruited from a specialized center located in Lima, the capital city of Peru, therefore, extrapolation of our results to other populations in Peru or Latin America is limited. Moreover, although the tool has not been validated for use in Latin America, it has been utilized as a functional assessment tool in Spanish-speaking populations in international clinical trials.^44^ The mean educational level of patients from Lima is higher compared with other regions of Peru, therefore, it is it is necessary to validate the ADCS-ADL in a population with low educational levels. In addition, the tool itself does not involve current events and is outdated to some degree. The COVID-19 pandemic has affected patients with neurodegenerative diseases,^45^ with many patients with dementia needing to quarantine in order to decrease their infection and morbidity risks. Patients with sufficient functional ability have come to increase their ability to rely on technological devices during the pandemic, which is insufficiently covered by the ADCS-ADL. This emphasizes the importance of validating modern scales or adapting older scales as technologies develop, such as the Technology - Activities of Daily Living Questionnaire^12,46^.

## Conclusions

In conclusion, our study has demonstrated that the ADCS-ADL is an appropriate functional assessment tool for patients with a diagnosis of AD attending a specialized neurology clinic in Lima, Peru. Future work may serve to validate or adapt a functional assessment tool for the Peruvian context and accounts for low educational levels and technologies used in day-to-day life.

## References

[1] Dubois B Villain N Frisoni GB , et al. Clinical diagnosis of Alzheimer’s disease: Recommendations of the International Working Group. Lancet Neurol. 2021;20(6):484-496. DOI: 10.1016/S1474-4422(21)00066-1 33933186 PMC8339877

[2] Albert MS DeKosky ST Dickson D , et al. The diagnosis of mild cognitive impairment due to Alzheimer’s disease: Recommendations from the National Institute on Aging‐Alzheimer’s Association workgroups on diagnostic guidelines for Alzheimer's disease. Alzheimer’s Dementia. 2011;7:270-279. 10.1016/j.jalz.2011.03.008 PMC3312027 21514249

[3] Sánchez‐Benavides G Salvadó G Arenaza‐Urquijo EM , et al. Quantitative informant‐ and self‐reports of subjective cognitive decline predict amyloid beta PET outcomes in cognitively unimpaired individuals independently of age and APOE. Alzheimer’s Dementia: Diagnosis, Assessment & Disease Monitoring. 2020;12:e12127. doi:10.1002/dad2.12127 PMC7656171 33204815

[4] Jutten RJ Dicks E Vermaat L , et al. Impairment in complex activities of daily living is related to neurodegeneration in Alzheimer's disease-specific regions. Neurobiol Aging. 2019;75:109-116. 30557769 10.1016/j.neurobiolaging.2018.11.018

[5] Suzuki Y Teruya K Mochizuki H Nagasawa A Kondo T Shimoda N . Evaluation of activities of daily living/instrumental activities of daily living to accurately determine severity of moderate and severe Alzheimer’s disease: comparison of assessments by receiver operating characteristic curve and discriminant analyses. Dementia and Geriatric Cognitive Disorders Extra. 2019;9:227-235. doi:10.1159/000500019 31275348 PMC6600026

[6] Yi Y Ding L Wen H Wu J Makimoto K Liao X . Is Barthel Index suitable for assessing activities of daily living in patients with dementia? Front Psychiatr. 2020;11:282. doi:10.3389/fpsyt.2020.00282 PMC7225343 32457659

[7] Gordon MF Lenderking WR Duhig A , et al. Development of a patient‐reported outcome instrument to assess complex activities of daily living and interpersonal functioning in persons with mild cognitive impairment: The qualitative research phase. Alzheimer's Dementia. 2016;12:75-84. 10.1016/j.jalz.2015.04.008 26079412

[8] Marshall GA Sikkes SAM Amariglio RE , et al. Instrumental activities of daily living, amyloid, and cognition in cognitively normal older adults screening for the A4 study. Alzheimer's Dementia. 2020;12:e12118. DOI: 10.1002/dad2.12118 PMC7596668 33163609

[9] Quiroga LP Albala BC Klaasen PG . Validación de un test de tamizaje para el diagnóstico de demencia asociada a edad, en Chile. Rev Med Chile. 2004;132:467-478. doi:10.4067/s0034-98872004000400009 15382519

[10] Pfeffer RI Kurosaki TT Harrah CH Chance JM Filos S . Measurement of functional activities in older adults in the community. J Gerontol. 1982;37:323-329. 7069156 10.1093/geronj/37.3.323

[11] Lawton MP Brody EM . Assessment of Older People: Self-Maintaining and Instrumental Activities of Daily Living. Gerontol. 1969;9:179-186. 5349366

[12] Muñoz-Neira C López OL Riveros R Núñez-Huasaf J Flores P Slachevsky A . The technology - activities of daily living questionnaire: a version with a technology-related subscale. Dement Geriatr Cognit Disord. 2012;33:361-371. doi:10.1159/000338606 22797087 PMC4722866

[13] Cintra FCMDC Cintra MTG Nicolato R , et al. Functional decline in the elderly with MCI: Cultural adaptation of the ADCS-ADL scale. Rev Assoc Med Bras. 1992;63(7):590-599. 10.1590/1806-9282.63.07.590 28977084

[14] Galasko D Bennett D Sano M , et al. An inventory to assess activities of daily living for clinical trials in Alzheimer’s disease. The Alzheimer’s disease cooperative study. Alzheimer Dis Assoc Disord. 1997;11(suppl 2):S33-S39. 9236950

[15] Vogel A Bhattacharya S Waldorff FB Waldemar G . Proxy-rated quality of life in Alzheimer's disease: a three-year longitudinal study. Int Psychogeriatr. 2012;24:82-89. doi:10.1017/S1041610211001128 21729415

[16] Global Health Data Exchange (GHDx) . GBD Results Tool. Global Burden of Disease. http://ghdx.healthdata.org/gbd-results-tool (2019).

[17] Urrunaga-Pastor D Chambergo-Michilot D Runzer-Colmenares FM Pacheco-Mendoza J Benites-Zapata VA . Prevalence of cognitive impairment and dementia in older adults living at high altitude: A systematic review and meta-analysis. Dement Geriatr Cognit Disord. 2021;50:124-134. doi:10.1159/000514471 34139687

[18] Custodio N Montesinos R Lira D , et al. Validation of the RUDAS for the Identification of Dementia in Illiterate and Low-Educated Older Adults in Lima, Peru. Front Neurol. 2020;11:374. doi:10.3389/fneur.2020.00374 32477248 PMC7232574

[19] Sánchez SS Abanto J Sanchez-Boluarte A , et al. Frequency and associated factors of amnestic mild cognitive impairment at four senior citizen clubs in Lima, Peru. Dementia & Neuropsychologia. 2019;13:321-328. doi:10.1590/1980-57642018dn13-030009 31555405 PMC6753901

[20] McKhann GM Knopman DS Chertkow H , et al. The diagnosis of dementia due to Alzheimer’s disease: Recommendations from the National Institute on Aging‐Alzheimer’s Association workgroups on diagnostic guidelines for Alzheimer's disease. Alzheimer’s Dementia. 2011;7:263-269. doi:10.1016/j.jalz.2011.03.005 PMC3312024 21514250

[21] Sperling RA Aisen PS Beckett LA , et al. Toward defining the preclinical stages of Alzheimer’s disease: Recommendations from the National institute on aging‐Alzheimer’s association workgroups on diagnostic guidelines for Alzheimer’s disease. Alzheimer's Dementia. 2011;7:280-292. 10.1016/j.jalz.2011.03.003 PMC3220946 21514248

[22] Morris JC . Clinical dementia rating: a reliable and valid diagnostic and staging measure for dementia of the Alzheimer type. Int Psychogeriatr. 1997;9(1):173-176. doi:10.1017/s1041610297004870 9447441

[23] Morris JC Ernesto C Schafer K , et al. Clinical Dementia Rating training and reliability in multicenter studies. Neurology. 1997;48:1508-1510. 9191756 10.1212/wnl.48.6.1508

[24] Custodio N Lira D . Adaptación peruana del minimental state examination (MMSE). An Fac Med. 2014;75(1):69.

[25] Custodio N García A Montesinos R Lira D Bendezú L . Validación de la prueba de dibujo del reloj - versión de Manos - como prueba de cribado para detectar demencia en una población adulta mayor de Lima, Perú. Rev Peru Med Exp Salud Pública. 2011;28(1):29-34. 21537766 10.1590/s1726-46342011000100005

[26] Vega-Dienstmaier J Coronado-Molina Ó Mazzotti G . Validez de una versión en español del Inventario de Depresión de Beck en pacientes hospitalizados de medicina general. Rev Neuro Psiquiatr. 2014;77(2):95-103.

[27] Custodio N Becerra-Becerra Y Becerra-Becerra Y , et al. Validación y precisión de la escala de deterioro global (GDS) para establecer severidad de demencia en una población de Lima. CES Medicina. 2017;31(1):14-26.

[28] Custodio N Lira D Montesinos R Gleichgerrcht Manes F . Utilidad del Addenbrookes’s Cognitive Examination versión en español en pacientes peruanos con enfermedad de Alzheimer y demencia frontotemporal. Vertex Rev Arg de Psiquiat. 2012;XXIII:165-172. 23145370

[29] Rosen WG Mohs RC Davis KL . A new rating scale for Alzheimer's disease. Am J Psychiatr. 1984;141:1356-1364. 6496779 10.1176/ajp.141.11.1356

[30] Pascual LF Saz P Larumbe R , et al. Standardization of the Alzheimer's disease assessment scale in a spanish population. Neurologia. 1997;12:238-244. 9303590

[31] Rey A . L’examen psychologique dans les cas d’encéphalopathie traumatique (Les problems). Arch Psychol. 1941;28:286-340.

[32] Wechsler D . WAIS-III: Wechsler Adult Intelligence Scale. 3rd ed. San Antonio, TX: Psychological Corporation; 1997.

[33] Partington JE Leiter RG . Partington’s pathways test. Psychological Service Center Bulletin. 1949;1:9-20.

[34] Kaplan E Goodglass H Weintraub S . The Boston Naming Test. 2nd ed. Philadelphia, PA: Lea & Febiger; 1983.

[35] Nelson HE . A modified card sorting test sensitive to frontal lobe defects. Cortex. 1976;12(4):313-324. 1009768 10.1016/s0010-9452(76)80035-4

[36] Cummings JL Mega M Gray K Rosenberg-Thompson S Carusi DA Gornbein J . The Neuropsychiatric Inventory: Comprehensive assessment of psychopathology in dementia. Neurology. 1994;44:2308-2308. 7991117 10.1212/wnl.44.12.2308

[37] Fillenbaum GG . Screening the Elderly. J Am Geriatr Soc. 1985;33(10):698-706. 4045087 10.1111/j.1532-5415.1985.tb01779.x

[38] Souza RKMd. Barboza AF Gasperin G Garcia HDBP Barcellos PM Nisihara R . Prevalence of dementia in patients seen at a private hospital in the Southern Region of Brazil. Einstein (São Paulo). 2019;18:eAO4752. doi:10.31744/einstein_journal/2020AO4752 31664323 PMC6896655

[39] Perneczky R Wagenpfeil S Komossa K Grimmer T Diehl J Kurz A . Mapping scores onto stages: mini-mental state examination and clinical dementia rating. Am J Geriatr Psychiatr. 2006;14:139-144. doi:10.1097/01.JGP.0000192478.82189.a8 16473978

[40] Ard MC Galasko DR Edland SD . Improved statistical power of Alzheimer clinical trials by item-response theory: Proof of concept by application to the activities of daily living scale. Alzheimer Dis Assoc Disord. 2013;27:187-191. doi:10.1097/WAD.0b013e318265bcc1 22874658 PMC4362713

[41] Brennan L Siderowf A Rubright JD , et al. Development and initial testing of the penn parkinson’s daily activities questionnaire. Mov Disord. 2016;31:126-134. doi:10.1002/mds.26339 26249849 PMC4724261

[42] Raskin J Cummings J Hardy J Schuh K Dean R . Neurobiology of Alzheimer’s disease: integrated molecular, physiological, anatomical, biomarker, and cognitive dimensions. Curr Alzheimer Res. 2015;12:712-722. doi:10.2174/1567205012666150701103107. PMID: 26412218 PMCID: PMC5384474. 26412218 PMC5384474

[43] Guo HJ Sapra A . Instrumental Activity of Daily Living. In: StatPearls Treasure Island (FL): StatPearls Publishing; 2021. 31985920

[44] Winblad B Grossberg G Frolich L , et al. IDEAL: a 6-month, double-blind, placebo-controlled study of the first skin patch for Alzheimer disease. Neurology. 2007;69:S14-S22. doi:10.1212/01.wnl.0000281847.17519.e0 17646619

[45] Chambergo-Michilot D Barros-Sevillano S Rivera-Torrejón O De la Cruz-Ku GA Custodio N . Factors associated with COVID-19 in people with Parkinson’s disease: A systematic review and meta-analysis. Eur J Neurol. 2021;28:3467-3477. doi:10.1111/ene10.1111/ene.14912 33983673 PMC8239569

[46] IdiÃ!`quez J Torres F Madrid E Vega J Slachevsky A . Cuestionario de actividades de la vida diaria (T-ADLQ): utilidad en pacientes con accidente cerebrovascular menor. Rev MÃcopyrightdica Chile. 2017;145:188-193. 10.4067/S0034-98872017000200006 28453585

